# Effect of music therapy on anxiety and depression in breast cancer patients: systematic review and meta-analysis

**DOI:** 10.1038/s41598-024-66836-x

**Published:** 2024-07-17

**Authors:** Zhihui Xu, Cong Liu, Wenjun Fan, Shufan Li, Yuzhang Li

**Affiliations:** 1 School of Physical Education, Shanghai University of Sport, Shanghai, China; 2 School of Music, University of Leeds, Leeds, UK

**Keywords:** Music therapy, Cancer, Anxiety, Depression, Breast cancer, Gynaecological cancer

## Abstract

To systematically evaluate the intervention effect of music therapy on anxiety and depression in breast cancer patients. Randomised controlled trial (RCT) on music therapy for anxiety and depression in breast cancer patients was searched from 7 major databases, PubMed, Embase, the Cochrane Library, WOS, CNIC, Wanfang, and Wipro, spanning the period of library construction to 23 October 2023, and the literature screening of music therapy for anxiety or depression in breast cancer patients was carried out by 2 experimentalists, each of whom conducted a literature screening RCT independently of the other anxiety or depression in a RCT. Methodological quality was evaluated using the PEDro scale; GRADE profiler software for quality of evidence; and RevMan 5.4 was used for effect size merging and forest plots; publication bias tests and sensitivity analyses were performed using Stata 17.0; and standardized mean difference (SMD) and 95% CI were used as the effect statistics. A total of 13 RCTs with 1326 subjects (aged 18–70 years) were included in the literature, with a mean PEDro score of 6.8, and the literature was overall of good methodological quality. Meta-analysis showed that music therapy improved anxiety in breast cancer patients (841 cases), with a combined effect size (SMD = − 0.82, 95% CI [− 1.03, − 0.61] and *P* < 0.001); and improved depression in breast cancer patients (387 cases) with a combined effect size (SMD = − 0.76, 95% CI [− 1.15, − 0.38], *P* < 0.001). Subgroup analyses showed that music intervention with off-site music (757 cases) and music choice of non-self-selected music (537 cases) had the best effect on anxiety improvement, with corresponding combined effect sizes (SMD = − 0.88, *P* < 0.001; SMD = − 0.83, *P* < 0.001), respectively; followed by an intervention length of < 30 min (589 cases), a frequency of 2 times/day (382 cases), and intervention period of 2–3 weeks (101 cases) had the best effect on anxiety improvement, and the corresponding combined effect sizes were (SMD = − 0.80, *P* < 0.001; SMD = − 0.91, *P* < 0.001; SMD = − 1.02, *P* < 0.001), respectively; and the music selection was the choice of one's own favourite music among the expert recommendations (219 cases) (270 cases) had the best effect on the improvement of depressed mood, with combined effect sizes of (SMD = − 1.15, *P* < 0.001; SMD = − 0.71, *P* < 0.001) and music with an intervention duration of 30 min (287 cases), an intervention frequency of 1 time/day (348 cases), and an intervention period of 2–4 weeks (120 cases), respectively, with corresponding combined effect sizes of (SMD = − 0.75, *P* < 0.001; SMD = − 0.86, *P* < 0.001; SMD = − 1.06, *P* < 0.001), respectively. Music therapy can improve anxiety and depression in breast cancer patients, and the level of evidence is moderate. Although the heterogeneity between studies is high, which may lead to bias in the results, we explored the source of heterogeneity through subgroup and sensitivity analyses, providing a good evidence-based basis for clinical practice. The heterogeneity of anxiety and depression was explored by subgroup analysis, with anxiety due to music duration and music cycle; and depression due to intervention cycles and music duration. Sensitivity analyses also identified music duration and music cycle as contributing to the heterogeneity. Also, this study has some limitations since the included literature did not take into account the duration of the disease, education, and family economic status and did not categorize the age stages. This study found that music therapy improves anxiety and depression in breast cancer patients and the results can be used as a basis for clinical practice and researcher enquiry. This research has been registered on the INPLASY platform (https://inplasy.com/contact/) under the number: INPLASY2023100057.

## Introduction

The incidence of female breast cancer often ranks first among female malignant tumours^1^, accounting for 7–10% of all malignant tumors in the body. In the past 20 years, the incidence of breast cancer in China has increased by 37.6%, with an average annual growth rate of 2.3%^2^. Breast cancer patients are the phenomenon of uncontrolled proliferation of breast epithelial cells under the action of multiple carcinogenic factors; physiologically, the early stage often manifests symptoms such as breast lumps and enlarged axillary lymph nodes, and in the late stage, due to distant metastasis of the cancer cells, multi-organ lesions may occur, and even threaten the patients' lives^3^. Patients need to endure the pain and side effects of chemotherapy during treatment, and psychologically they often show emotional instability, anxiety depression, etc., and most of them have psychological problems such as moderate and severe anxiety levels.

Music therapy is an emerging psychotherapeutic modality in which the unique physiological and psychological effects of music enable patients to regulate their psychological disorders through musical experience and distraction. Music therapy is considered to be one of the most effective psychotherapeutic methods for clinical non-pharmacological treatment to eliminate psychosomatic disorders^4^. Music can affect patients' heart rate, blood pressure, respiratory rate, and blood cortisol levels through rhythm and tone^5^, thus relieving emotions such as anxiety or depression^6^. A study by Zhi Yanhong et al. found that patients use the sonic vibrations of music to produce beneficial resonance with certain physiological structures of the human body and achieve the elimination of psychological barriers^7^; a study by LAGATTOLLA F et al. ^8^ showed that music can promote relaxation in patients with breast cancer, lower anger, heart rate, respiratory rate, reduce pain, reduce the need for anesthesia, analgesia^9^ and other recourse, and shorten the recovery time, thus reducing anxiety and depression^10^.

Previous research has shown that music therapy can effectively improve anxiety and depression symptoms in breast cancer patients^11,12^, but the specific intervention protocol is unclear, especially the choice of music, intervention duration, intervention period, and intervention frequency need to be further clarified. The results of some of the previous research are controversial, such as studies found that the duration of music intervention is most effective when it is less than 30 min, and some research has also shown that the longer the duration, the better the effect. Based on this, this research intends to further explore the effects of music selection, intervention mode, intervention duration, intervention period, and intervention frequency on anxiety and depression in breast cancer patients based on previous studies, to provide precise music intervention programs for clinics and references for researchers.

## Methods

### Literature search strategy

This study was reported in accordance with PRISMA (Preferred Reporting Items for Systematic Evaluation and Meta-Analysis) guidelines to ensure transparency of the research^13^. The research protocol was registered on INPLASY No.INPLASY2023100057.

Data sorting and statistical analysis were conducted for the included literature in accordance with the requirements of the International Guidelines for Writing Systematic Reviews^14^. The two researchers independently computer-searched six databases from China Knowledge, Wanfang, PubMed, Embase, the Cochrane Library, and Web of Science for RCTs of music therapy for breast cancer patients. The search date of each database was 23 October 2023. The search was conducted by combining subject terms with free words, using the Boolean symbols "AND" and "OR" for combinatorial concatenation, and was determined after repeated pre-testing. In case of disagreement between two researchers, the decision was discussed with the third researcher. This was supplemented by tracking down the relevant systematic reviews and references of the included literature. Table 1.

**Table 1:** Literature search steps.

Database	search step
PubMe and The Cochrane Libraryhe retrieval strategy	#1 “Therapy, Music”[Mesh]OR “Music”[Title/Abstract]OR “Timbre”[Title/Abstract] OR “Music therapy” [Title/Abstract] #2 “breast cancer” [Mesh] OR “breast neoplasms” [Title/Abstract] OR “breast carcinoma” [Title/Abstract] OR “breast” [Title/Abstract] OR “tumor” [Title/Abstract] #3 “anxiety” [Mesh] OR “Depression” [Title/Abstract] OR “psychology” [Title/Abstract] #4 Randomized controlled trial[Publication Type] OR “Randomized” [Title/Abstract] OR “controlled”[Title/Abstract] OR “Trial” [Title/Abstract] #5 #1 AND #2 AND #3 AND #4
Web of Science retrieval strategy	#1 TS = (“Therapy, Music” OR “Music” OR “Timbre” OR “Music therapy”) #2 TS = (“breast cancer” OR “breast neoplasms” OR “breast carcinoma” OR “breast” OR “tumor”) #3 TS = (“anxiety” OR “Depression” OR “psychology”) #4 TS = (“Randomized controlled trial” OR “Randomized” OR “Controlled” OR “Trial”) #5 #1 AND #2 AND #3 AND #4
Embase retrieval strategy	#1 “Therapy,Music”[exp]OR “Music”[ab,ti]OR “Timbre”[ab,ti] OR “Music therapy” [ab,ti] #2 “breast cancer”[exp] OR “breast neoplasms” [ab,ti] OR “breast carcinoma” [ab,ti] OR “breast ” [ab,ti] OR “tumor” [ab,ti] #3 “anxiety” [exp] OR “Depression” [ab,ti] OR “psychology”[ab,ti] #4 “Randomized controlled trial” [exp] OR “Randomized” [ab,ti] OR “Controlled” [ab,ti] OR “Trial” [ab,ti] #5 #1 AND #2 AND #3 AND #4
CNIC retrieval strategy	(music therapy + music + tones + pieces + non-pharmacological treatments) and (breast cancer + breast cancer patients + breast tumours + breast tumours) and (anxiety + depression + emotional + psychological)
Wan fang retrieval strategy	(music therapy or music or non-pharmacological therapy or tone or music) and (breast cancer or breast cancer patients or breast tumours or breast tumours) and (anxiety or depression or emotion or psychology)

### Inclusion criteria

This research is based on the ICD and ACJJ classification system to construct a PICO framework for systematically evaluating and analysing the intervention effect of music therapy on anxiety and depression in breast cancer patients, and the research shows that the intervention effect of music therapy on anxiety and depression in breast cancer patients is significant. Table 2.

**Table 2:** PICOS strategy—inclusion criteria.

PICOS	Inclusion criteria
Research target	Compliance with the International Classification of Diseases(International Classification of Disease,ICD)-10 and the American Joint Committee on Cancer(American Joint Committee on Cancer, AJCC)Anyone of the diagnostic criteria
Intervention	The experimental group intervention was music therapy
Comparison	The control group was treated conventionally
Outcome indicator	Mood-related indicators: 1. anxiety 2. depression
Type of research	RCT

*P* Population, *I* Intervention, *C* Comparison, *O* Outcome, *S* Study design.

### Exclusion Criteria

(1) Literature with repeated publications or PEDro score < 4. (2) No detailed data is provided, and specific data cannot be obtained. (3) The experimental group received a combination intervention, such as music therapy combined with aerobic exercise.

### Data extraction

After retrieving the relevant literature, the literature was imported into Endnote for weight removal. Literature screening and data extraction were performed by 2 researchers using an independent double-blind approach respectively. The extracted data from eligible studies were entered into RevMan 5.4 and double-checked for accuracy, and in case of disagreement, the 3rd researcher joined the discussion to decide whether to include them or not. The extraction included basic information about the literature (first author, year of publication), basic information about the experimental subjects (sample size, age, gender), music therapy (classical Chinese folk music, world-famous music, soft, beautiful tracks, etc.), testing methods, specific outcome indicators: anxiety and depression, and extraction of baseline and posttest data.

### Quality evaluation

The quality of the literature was evaluated using a modified version of the PEDro scale^15^ to assess the methodological quality of the included literature. The scale included 10 criteria: ITT intention-to-treat analysis, random allocation, allocation concealment, baseline similarity, blinding of the research subjects, blinding of the outcome assessment, dropout rate ≤ 15%, intention-to-treat analysis, blinding of the therapists, as well as the point measure and the discrepancy measure. A total of 10 points was assigned to the scale, with a score of less than 4 considered poor quality, 4 to 5 considered moderate quality, 6 to 8 considered good quality, and 9 to 10 considered high quality, and only literature with a score of 5 or higher was included.

Quality of evidence evaluation^16^ was done through GRADE profiler software, and the quality of evidence for outcome indicators was evaluated on five downgraded factors including publication bias, inconsistency, imprecision, indirectness, and limitations of the research. Among them, a downgrade of 3 was considered as very low-level evidence, a downgrade of 2 was considered as low-level evidence, a downgrade of 1 was considered intermediate-level evidence, and no downgrade was considered as high-level evidence, and the final grade of evidence was categorized into 4 grades: high level, intermediate level, low level, and very low level. Quality ratings were conducted independently by two researchers, with a third researcher contributing to the discussion if there was disagreement.

### Statistical methods

Review Manager 5.4 was used to statistically analyze the data. The outcome indicators of the literature included in this paper are all continuous variables, and the measurement tools are inconsistent across research, so the effect indicators were calculated using the effect size (Standard Mean Difference (SMD)); when the effect size is < 0.2, it is a small effect, 0.20–0.49 is a medium-small effect, 0.50–0.79 is a medium effect, and ≥ 0.8 is a large effect^17^. Heterogeneity was quantified objectively by I^2^, and heterogeneity was quantified by the I^2^ statistic. 75%, 50%, and 25% were the boundary values of high, medium, and low heterogeneity, respectively^18^.If there was no statistical heterogeneity among the results, the fixed effect model was used. If heterogeneity exists, the random effects model is used to combine the effect sizes. And the Stata 17.0 Egger test is used to publication bias. If there is publication bias, the scission-supplement method is used to correct it.

## Results

### Literature search results

**Figure 1:**
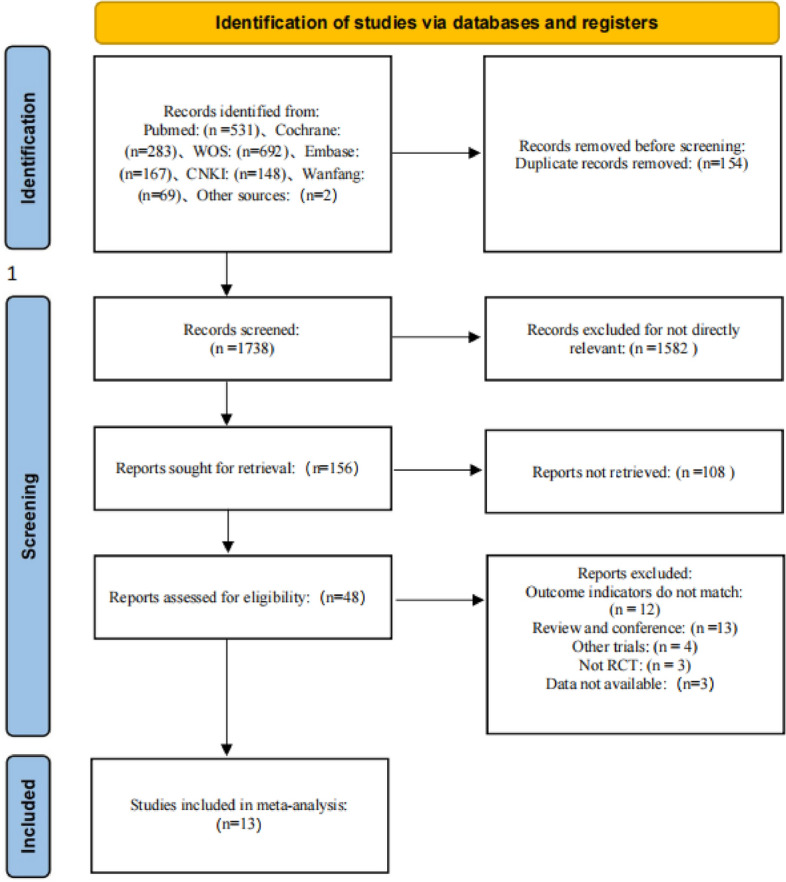
Document retrieval flow chart.

As shown in Fig. 1, a total of 1738 pieces of literature were retrieved through PubMed, Embase, Web of Science, Cochrane, CNKI, Wanfang, and VIP databases, and 48 pieces of literature were selected after preliminary screening by eliminating duplicates, reading titles, and abstracting. According to the inclusion and exclusion criteria, the full text was re-screened, and finally 13 articles were included in the meta-analysis.

### Basic features of the included literature

The 13 articles^19–31^ included were published between 2006 and 2022, with a total of 1326 cancer patients. The intervention content of the experimental group was music therapy combined with conventional intervention, and the control group was conventional chemotherapy intervention (Table 3).

**Table 3:** Basic characteristics of included studies (*n* = 13).

Name	Sample size	Age (years)		Characteristics of the intervention					Outcome
	(T/C)	T	C	Time (min)	Exercise program duration (week)	Frequency (sessions/week)	Expert Recommendations(Yes/No)	Music Selection	
Li et al. 2012^19^	54/51	44 .88 ± 9 .37	45.13 ± 9.48	30	4	2	Yes	Choose by oneself	1
Chirico et al. 2019^20^	30/34	18–70	18–70	20	–	–	Yes	Other person’s choice	1
Lima et al. 2020^21^	16/17	49.50 ± 10.65	50.76 ± 9.45	30	18	2	Yes	Other person’s choice	5, 6
Hanser et al. 2006^22^	18/22	26–74	36–77	45	12	3	Yes	Other person’s choice	11
Palmer et al. 2015^23^	65/62	58.2 ± 17.6	61.3 ± 14.9	5	3	1	Yes	Other person’s choice	7
Zhou et al. 2011^24^	60/60	44 .88 ± 9 .37	45.13 ± 9.48	30	4	2	Yes	Choose by oneself	8
Shu-Xian et al. 2013^25^	40/40	Over 18 years old	Over 18 years old	60	6	2	No	Other person’s choice	3, 2, 4, 9
Zhen-Qi et al. 2010^26^	52/50	Over 18 years old	Over 18 years old	30	6	2	No	Other person’s choice	1
Fu et al. 2009^27^	40/38	26.3 ~ 60.9	39.3	40 ~ 60	4	2	No	Other person’s choice	2, 3
Chang et al. 2022^28^	30/30	54.1 ± 5.2	56.3 ± 6.2	30	4	1	No	Choose by oneself	2, 3
Yuting et al. 2010^29^	158/152	39.3 ± 5.8	39.3 ± 5.8	–	1	–	No	Other person’s choice	2
Lin et al. 2010^30^	10/10	39.7 ± 13.2	37.2 ± 12.7	20	single	1	No	Other person’s choice	10
Hongmei et al. 2008^31^	40/40	44.2 ± 15.6	45.8 ± 14.9	30	single	1	Yes	Choose by oneself	1

T/C stands for experimental group/control group. Outcome indicators 1.SAI: State Anxiety Scale, 2.SAS: Self-rating Anxiety Scale, 3.SDS Self-rating Depression Scale, 4.HAMD: Hamilton Depression Scale, 5.BAI: Baker Anxiety Scale, 6.BDI-II: Baker Depression Scale, 7.GA-VAS: Visual Analog Scale, 8.ZSDS. HAMA: Hamilton Anxiety Scale 10.STAI: State-Trait Anxiety Scale 11.HADS: Hospital Anxiety and Depression Scale.

### Article quality evaluation

**Figure 2:**
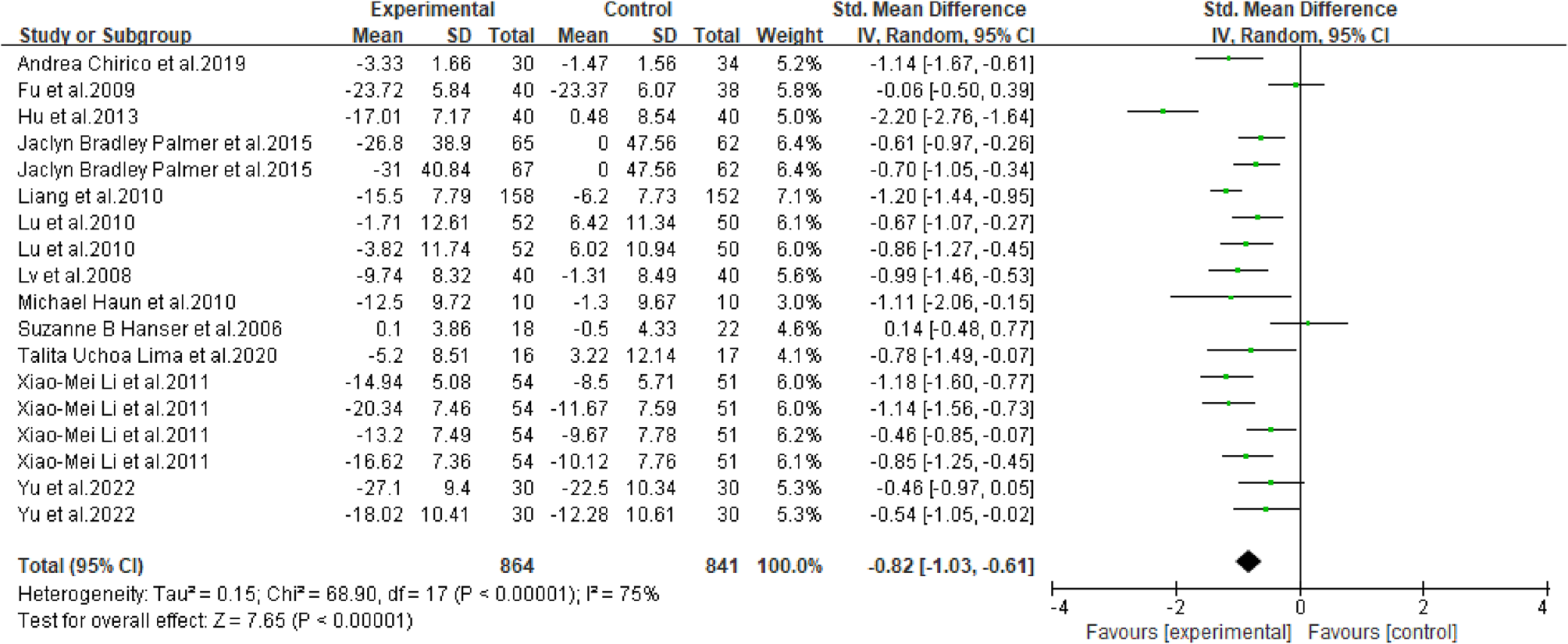
Forest map of anxiety in breast cancer patients with music therapy.

All studies described ITT intention-to-treat analyses, statistical analyses were performed between groups, point measures and difference-in-difference scales, random allocation with similar baselines, dropout rates ≤ 15%, five studies described allocation concealment, three studies blinded study participants, three studies blinded outcome assessments, and no studies blinded therapists. Of the 13 studies included, the PEDro scale scores ranged from 6 to 8, with a mean of 6.8, giving good overall research quality (Table 4) (Fig. 2).

**Table 4:** Methodological quality assessment included in the study (n = 13).

	Study	Random allocation	Allocation concealment	Baseline similarity	Blinding of research subjects	Therapists blind	Result evaluation blinding	Exit rate ≤ 15%	ITT Intention to Treat Analysis	Statistical analysis between groups	Point measurement and difference magnitude	Total score
1	Li et al. 2012^19^	1	0	1	0	0	0	1	1	1	1	6
2	Chirico et al. 2019^20^	1	0	1	1	0	1	1	1	1	1	8
3	Lima et al. 2020^21^	1	0	1	0	0	0	1	1	1	1	6
4	Hanser et al. 2006^22^	1	0	1	1	0	1	1	1	1	1	8
5	Palmer et al. 2015^23^	1	0	1	0	0	0	1	1	1	1	6
6	Zhou et al. 2011^24^	1	0	1	0	0	0	1	1	1	1	6
7	Shu-Xian et al. 2013^25^	1	1	1	0	0	0	1	1	1	1	7
8	Zhen-Qi et al. 2010^26^	1	1	1	0	0	0	1	1	1	1	7
9	Fu et al. 2009^27^	1	0	1	0	0	0	1	1	1	1	6
10	Chang et al. 2022^28^	1	1	1	0	0	0	1	1	1	1	7
11	Yuting et al. 2010^29^	1	1	1	0	0	0	1	1	1	1	7
12	Lin et al. 2010^30^	1	0	1	1	0	1	1	1	1	1	8
13	Hongmei et al. 2008^31^	1	1	1	0	0	0	1	1	1	1	7

1 represents meeting the entry, 0 represents not meeting the entry.

### Results of *meta*-analysis

#### Meta-analysis of music therapy on anxiety in breast *cancer* patients

As shown in Fig. 2, among the included literature, 12 studies (1278 patients) evaluated the effect of music therapy on anxiety in breast cancer patients. Heterogeneity test *I*^2^ = 75%, *P* < 0.01. There was a high degree of statistical heterogeneity among the studies, so the random effects model was used to combine the effect sizes. The results of the meta-analysis showed that the effect size was -0.82, 95%CI[-1.03,-0.61], and the difference between the experimental group and the control group was statistically significant. 0.001, indicating that music therapy was effective in reducing anxiety in breast cancer patients compared to the control group.

**Figure 3:**
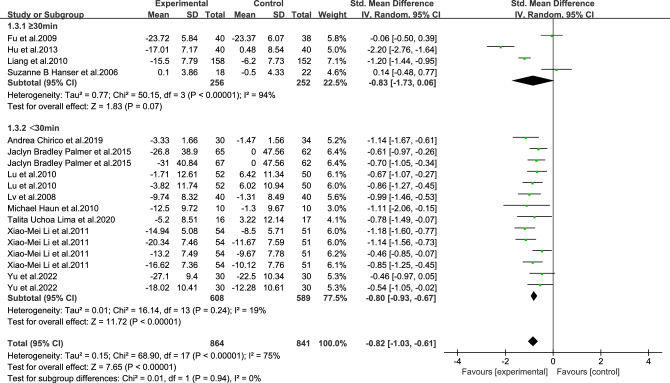
Subgroup analysis of the influence of different music duration on anxiety of breast cancer patients.

**Figure 4:**
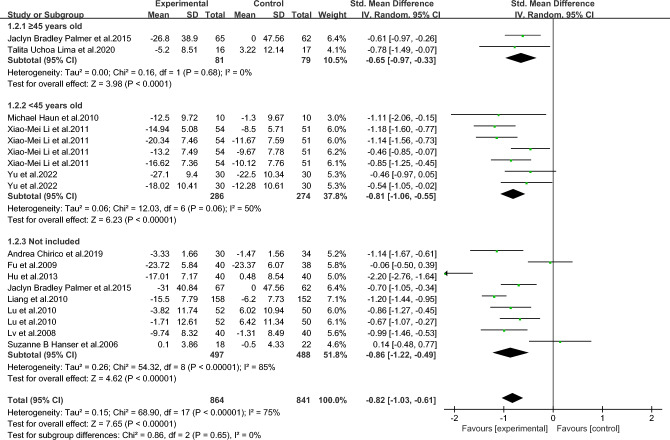
Subgroup analysis of influence of average age of patients on anxiety of breast cancer patients.

**Figure 5:**
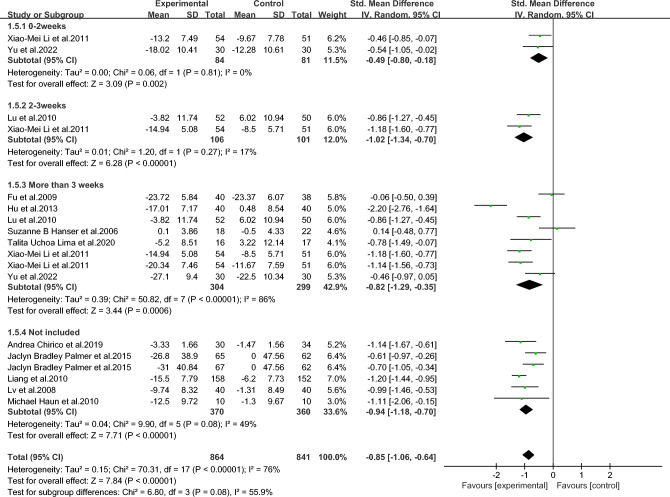
Subgroup analysis of the influence of different music intervention cycles on anxiety of breast cancer patients.

**Figure 6:**
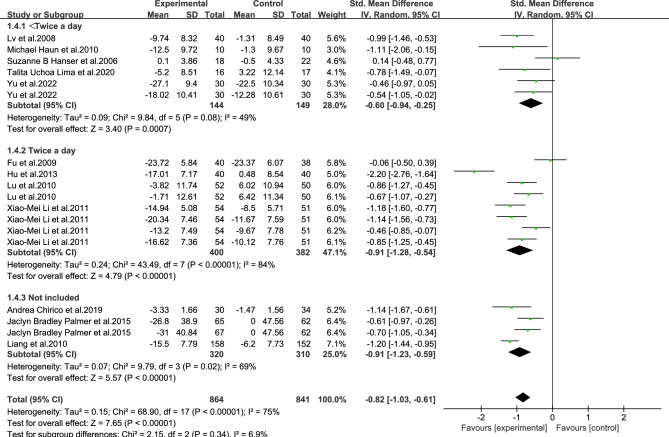
Subgroup analysis of the influence of music intervention frequency on anxiety of breast cancer patients.

**Figure 7:**
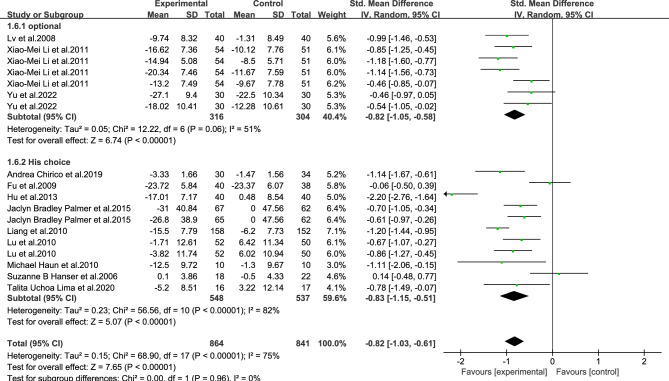
Subgroup analysis of the influence of different music choices on anxiety of breast cancer patients.

**Figure 8:**
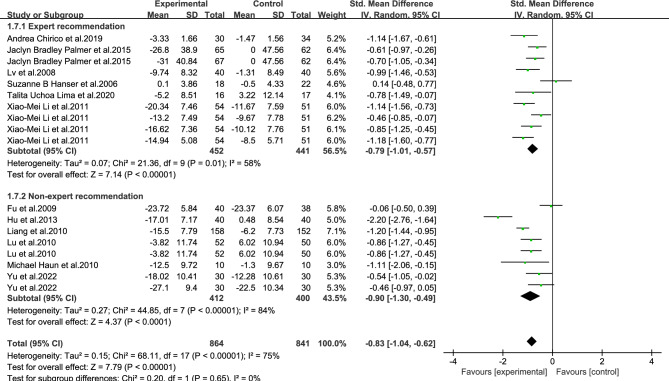
Is it a subgroup analysis of the influence of music recommended by experts on anxiety of breast cancer patients.

**Figure 9:**
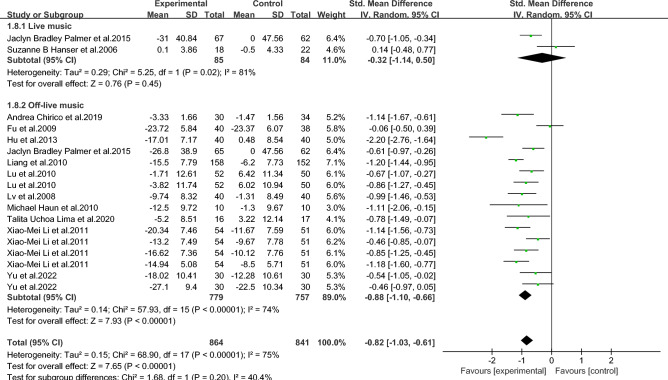
Subgroup analysis of the influence of different music intervention methods on anxiety of breast cancer patients.

As shown in Figs. 3, 4, 5, 6, 7, 8 and 9.In order to explore the source of heterogeneity, subgroup analysis was performed for the main outcome indicator, anxiety.The effect of music therapy on the anxiety of breast cancer patients may be influenced by factors such as average age, intervention duration, intervention cycle, intervention frequency, music selection, professional degree, and music style. The results of subgroup analysis showed that intervention cycle, intervention duration, intervention frequency, music selection, professionalism, period, average age and subgroup analysis of music style were statistically significant (*P* < 0.001). From the perspective of heterogeneity sources, intervention duration, average age, intervention period and intervention cycle may be the sources of heterogeneity (Table 5).

**Table 5:** Subgroup analysis of the effect of music therapy on anxiety in breast cancer patients.

Research characteristics	Group	Effect size		Heterogeneity	
		SMD(95%CI)	P	I^2^	P
Intervention duration	> 30 min	− 0.83(− 1.73,0.06)	0.07	94	< 0.001
	≤ 30 min	− 0.80(− 0.93,− 0.67)	< 0.001	19	0.24
Mean age	≥ 45 years old	− 0.65(− 0.97,− 0.33)	< 0.001	0	0.68
	< 45 years old	− 0.81(− 1.06,− 0.55)	< 0.001	50	0.06
	Not included	− 0.86(− 1.22,− 0.49)	< 0.001	85	< 0.001
Intervention cycle	0–2 weeks	− 0.49(− 0.80,− 0.18)	0.002	0	0.81
	2–3 weeks	− 1.02(− 1.34,− 0.70)	< 0.001	17	0.27
	More than 3 weeks	− 0.82(− 1.29,− 0.35)	< 0.001	86	< 0.001
	Not included	− 0.94(− 1.18,− 0.70)	< 0.001	49	0.08
Intervention frequency	< Twice a day	− 0.60(− 0.94,− 0.25)	< 0.001	49	0.08
	Twice a day	− 0.91(− 1.28,− 0.54)	< 0.001	84	< 0.001
	Not included	− 0.91(− 1.23,− 0.59)	< 0.001	69	0.02
Music selection	optional	− 0.82(− 1.05,− 0.58)	< 0.001	51	0.06
	His choice	− 0.83(− 1.15,− 0.51)	< 0.001	82	< 0.001
Degree of specialization	Expert recommendation	− 0.79(− 1.01,− 0.57)	< 0.001	58	0.01
	Non-expert recommendation	− 0.90(− 1.30,− 0.49)	< 0.001	84	< 0.001
Intervention mode	Live music	− 0.32(− 1.14,− 0.50)	0.45	81	0.02
	Off-live music	− 0.88(− 1.10,− 0.66)	< 0.001	74	< 0.001

#### Meta-analysis of depression in breast *cancer* patients with music therapy

**Figure 10:**
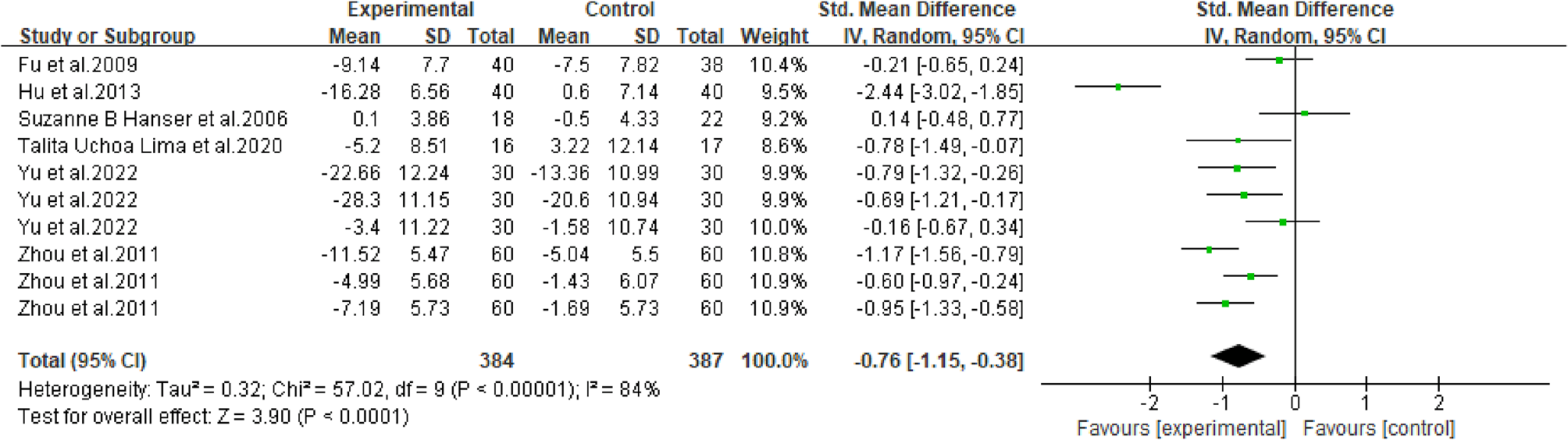
Music therapy for overall depression in breast cancer patients.

As shown in Fig. 10, six studies in the included literature evaluated the effect of music therapy on depression in breast cancer patients, including 411 patients. Heterogeneity test I2 = 84%, *P* < 0.01. There was a high degree of statistical heterogeneity among the studies, so the random effects model was used to combine the effect sizes. The results of meta-analysis showed that the effect size was -0.76, 95%CI[-1.15,-0.38], and the difference was statistically significant. 0.00001, indicating that music therapy was effective in alleviating depression in breast cancer patients compared to controls.

**Figure 11:**
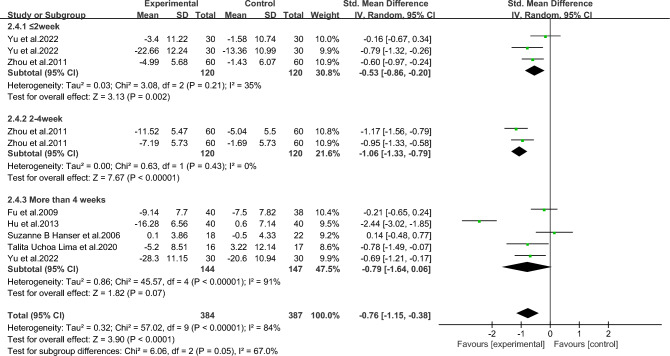
Subgroup analysis of the impact of different music intervention cycles on depression in breast cancer patients.

**Figure 12:**
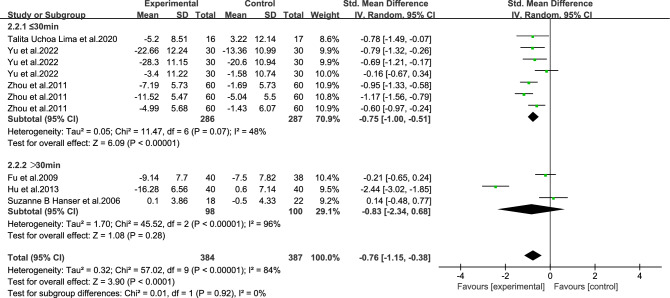
Subgroup analysis of the influence of different music intervention duration on depression in breast cancer patients.

**Figure 13:**
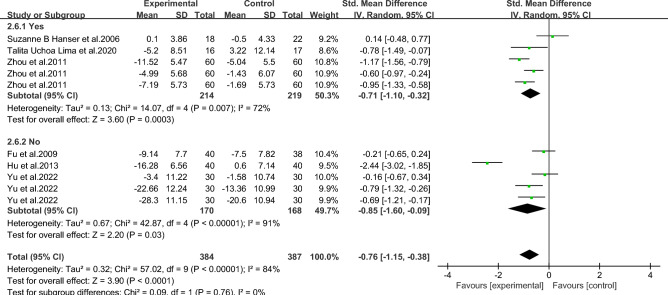
Subgroup analysis of whether experts recommend music for depression in breast cancer patients.

**Figure 14:**
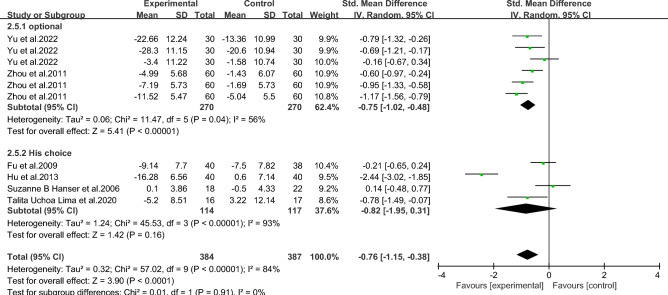
Subgroup analysis of the influence of music selection on depression in breast cancer patients.

**Figure 15:**
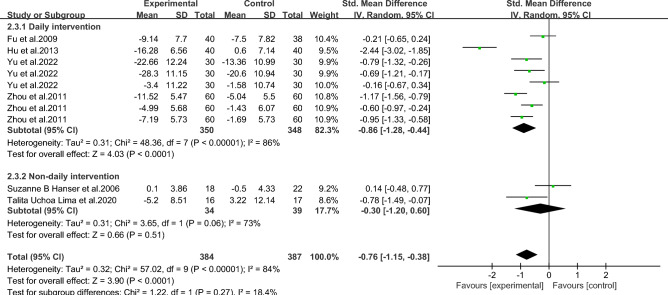
Subgroup analysis of the influence of music intervention frequency on depression in breast cancer patients.

As shown in Figs. 11, 12, 13, 14 and 15.To explore the source of heterogeneity, subgroup analysis was performed for depression, the main outcome indicator. The effect of music therapy on anxiety and depression in breast cancer patients may be limited by the intervention cycle, music duration, professional degree, music choice and frequency. The results of subgroup analysis in the table showed that intervention cycle, intervention duration, age specialization, music selection and subgroup analysis of group music style were statistically significant. From the source of heterogeneity, intervention cycle and duration may be the main source of heterogeneity(Table 6).

**Table 6:** Subgroup analysis of the effects of music therapy on depression in breast cancer patients.

Research characteristics	Group	Effect size		Heterogeneity	
		SMD (95%CI)	*P*	I^2^	*P*
Intervention cycle	≤ 2 Weeks	− 0.53(− 0.86,− 0.20)	0.002	35	0.21
	2–4Weeks	− 1.06(− 1.33,− 0.79)	< 0.001	0	0.43
	More than 4 weeks	− 0.79(− 1.64,− 0.06)	0.07	91	< 0.001
Intervention duration	≤ 30 min	− 0.75(− 1.00,− 0.51)	< 0.001	48	0.07
	> 30 min	− 0.83(− 2.34,− 0.68)	0.28	96	< 0.001
Degree of specialization	Yes	− 1.15(− 1.42,− 0.88)	< 0.001	95	< 0.001
	No	− 0.26 (− 0.53,0.01)	0.14	50	0.06
Music selection	Optional	− 0.71 (− 1.10,− 0.32)	< 0.001	72	0.007
	His choice	− 0.85 (− 1.60,− 0.09)	0.03	91	< 0.001
Intervention frequency	Daily intervention	− 0.86 (− 1.28,− 0.44)	< 0.001	86	< 0.001
	Non-daily intervention	− 0.30 (− 1.20,0.60)	0.51	73	0.06

### Risk *bias* analysis

**Figure 16:**
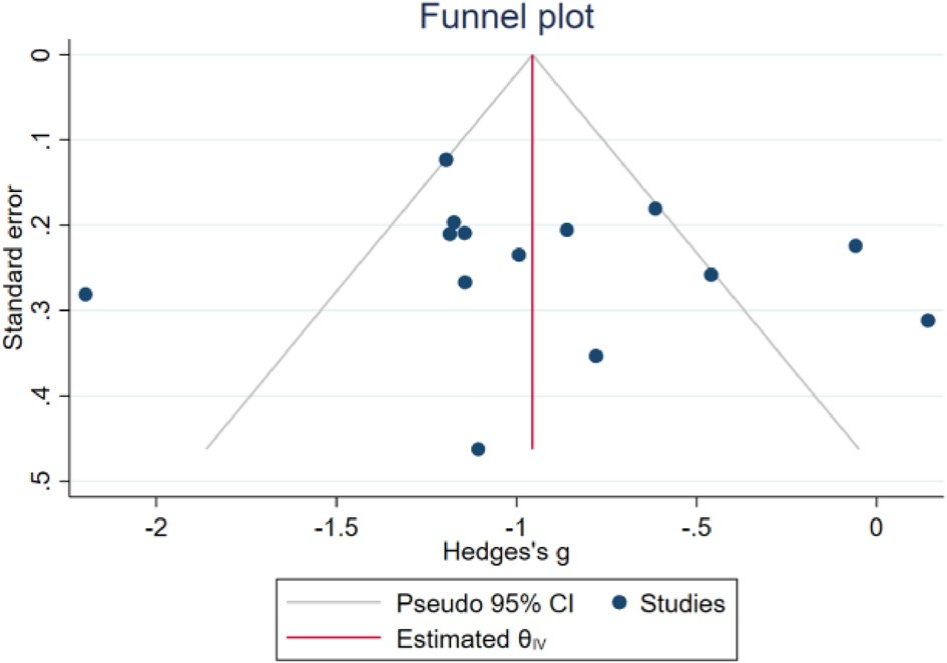
Music therapy for breast cancer patients funnel diagram.

As shown in Fig. 16, it can be found that the funnel diagram of music therapy on the anxiety of breast cancer patients is basically symmetric. The Egger test result shows that Z = − 0.22, P >|z|= 0.8224, indicating that there is no publication bias in the study.

### Sensitivity analyses

**Figure 17:**
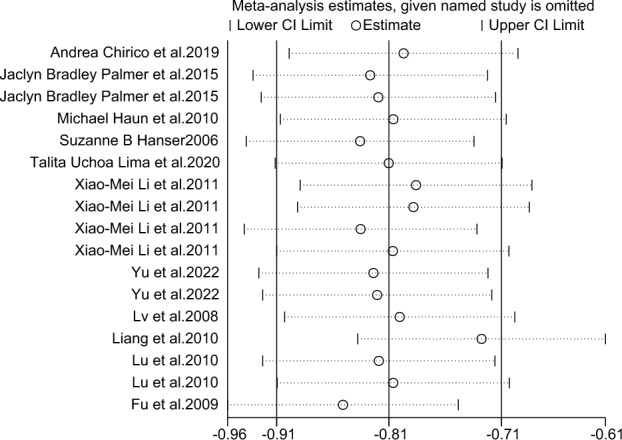
Sensitivity analysis of music therapy on anxiety in breast cancer patients.

As shown in Fig. 17, to investigate whether the heterogeneity between studies was caused by individual studies, the overall effects were analyzed by screening individual studies one by one. The normal value of the amplitude of the effect of music therapy on anxiety in breast cancer patients included in all studies was in the range (-0.91 to -0.71). The analysis results showed that the data sensitivity of this study was relatively low and did not fundamentally change the results of the meta-analysis, indicating that the research results have a certain stability and reliability.

### Evaluation of evidence quality level

**Figure 18:**
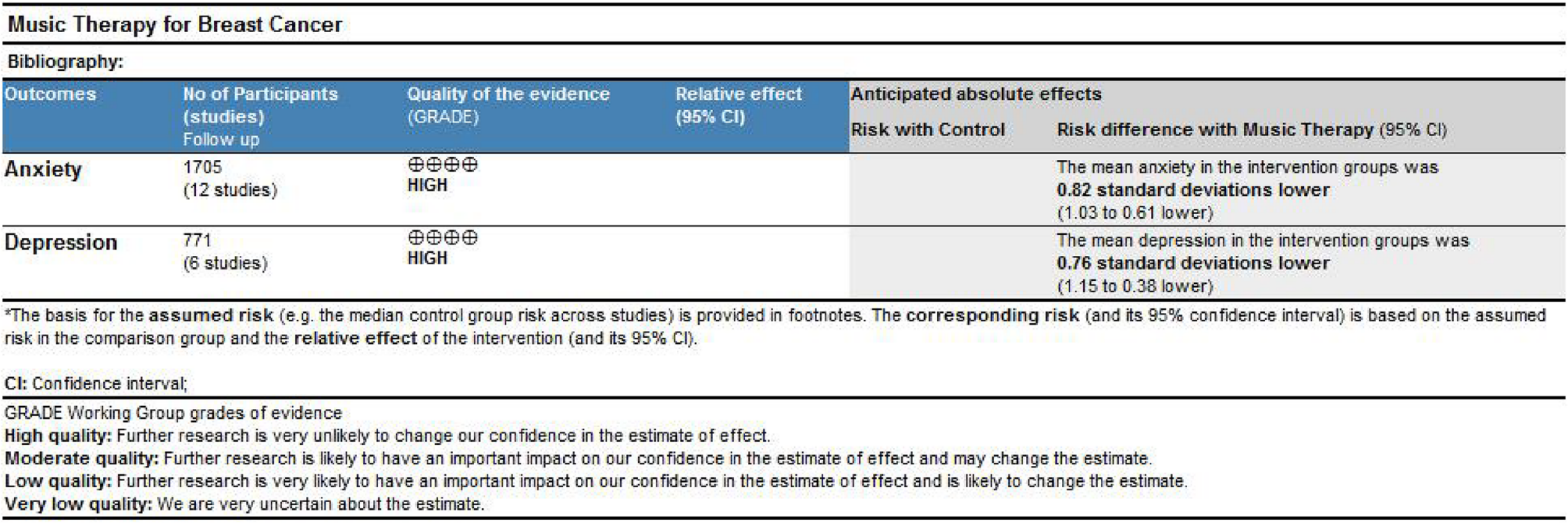
Quality of evidence for the effect of music therapy on improving anxiety and depression in breast cancer patients.

As shown in Fig. 18, the GRADEpro evidence rating system was used to assess the quality of evidence for the outcome indicators. It was found that the quality of evidence for music therapy for improving anxiety and depression in breast cancer patients was high, and the actual effect was close to the research findings. However, the heterogeneity of the articles is high, so we downgraded the quality of evidence to moderate.

### Adverse events

No adverse events resulting from music therapy were reported in any of the 13 included papers.

## Discussion

### Influence of music therapy on the anxiety of breast *cancer* patients

The results of this study show that music therapy can significantly improve anxiety in breast cancer patients, and the results are consistent with previous studies. Previous meta-analyses found that music therapy eliminated psychological barriers through unique effects. Longitudinal studies^21,32^ have found that music can reduce pain^33^and shift attention away from negative stimuli to things that are familiar, soothing, and pleasant^10,11^. It has also been confirmed that music therapy can reduce the anxiety and depression of patients with other diseases^34–39^.

A Total of 13 papers were included in this study to systematically evaluate the intervention effect of music therapy on anxiety and depression in breast cancer patients using the PEDro scale to evaluate the risk of bias of the included papers in 9 aspects, with a mean score of 6.8, which was found to be of good quality, but this study did not carefully classify the condition and type of the breast cancer patients in the included papers, and with an I^2^ > 50%, there was a high degree of heterogeneity among the studies and the existence of a considered some inconsistency. In the research, a dose–effect relationship was found for the effects of music therapy on anxiety and depression in breast cancer patients, which raised the level of evidence to one. Limitations of the research:1) The types of music selected for the interventions included in the literature were different, with no fixed criteria, whether it was recommended by the experts in English or selected by their favorite music, and the repertoire selected was more often than not classified specifically, which inspired the expectation of more high-quality RCT articles to be further developed. The study was conducted to identify the effects of music therapy on anxiety and depression in patients with breast cancer. RCT articles on music categorization to further complement and demonstrate. In conclusion, music therapy is given high-level quality of evidence for both anxiety and depression intervention effects in breast cancer patients.

This study found that breast cancer patients had the best improvement effect on anxiety when the music was their favorite music recommended by experts, the intervention method was non-live music, the intervention duration was less than or equal to 30 min, the intervention frequency was 2 times per day, and the intervention cycle was 2–3 weeks. Anxiety in breast cancer patients is mostly induced by low estrogen levels, and the decrease in estrogen levels is accompanied by a decline in serotonin transmitters. The possible mechanism by which music can improve anxiety in patients may be as follows: Music can increase the levels of estrogen^40^ and oxytocin^41^, and also reduce the concentration of serum cortisol in women, so that the value of serum cortisol in breast cancer patients can return to normal^42^. In terms of the nervous system, music can increase the dopamine activity of nucleus accumbens (NAc) and ventral tegmental cortex (VTA), alter the structural changes of the mesolimbic brain (e.g., nucleus accumbens [NAc], ventral tegmental area [VTA]), and effectively control the influence of emotional stimuli on the autonomic and physiological responses of the hypothalamus and insula^43^. Inhibition of sympathetic nervous system reactivity reduces negative emotional experiences^42^, thereby reducing anxiety. Choosing their favorite music has a better effect on breast cancer patients^44^ to perform non-live music, which may be because patients can get into the state more quickly when choosing their favorite music and local music^45^, so that patients can resonate the cranial cavity, chest cavity, or a certain tissue cavity through rhythm, frequency, and regular sound wave vibration^46^. It directly affects people's brain waves, heart rate, and breathing rhythm, thereby reducing anxiety. It may also be because, when listening to recorded music, patients can enter the music environment faster by restricting light, sound, visitors, and phone calls, thus regulating their emotional state^47^. A music intervention with a duration of no more than 30 min, a frequency of 2 times per day, and a cycle of 2–3 weeks is more effective for breast cancer patients. PALMER^23^also found that music therapy can reduce anxiety within only 5 min and can significantly reduce anxiety during 15–30 min of hypnotic intervention. Regarding the frequency of intervention, 2/day are statistically significant, and the reason remains unclearly, more high-quality RCTs need to complement and prove this in the future. The effect of music intervention on patients' anxiety may present an inverted "U" curve, and the intervention effect is the best at the hour, followed by more than 3 weeks, and the effect is not good within 2 weeks^6^.

This study also found that the duration of the intervention was inconsistent with that of Fu Yanzhi et al.^23,24,27^. The duration of music intervention did not become more effective over time, and the range of change from pre-treatment to post-treatment did not decrease over time. The reason might be that the patients studied by Fu Yanzhi and others were patients with advanced breast cancer, and this might also be related to the timeliness of behavioral habits. At the same time, it also brings inspiration to future generations to further explore the impact of music duration on anxiety in breast cancer patients and look forward to more high-quality RCT articles to further supplement and prove. We also found that different types of music (such as classical music, soothing music, and cheerful music) have different effects on anxiety in breast cancer patients, but the number of previous literature is too small to classify, which also needs more high-quality RCTs to verify.

### Influence of music therapy on depression in breast *cancer* patients

This study found that music selected as expertly recommended music, an intervention duration of 30 min, an intervention frequency of 1 time per day, and an intervention cycle of 2–4 weeks had the best effect on improving depression in breast cancer patients. This is consistent with previous results^11,12^. Longitudinal studies^48^ have found that music can stimulate the cerebral cortex in many ways, evoking pleasant thought associations and emotions in patients.

Patients with breast cancer are more prone to depression symptoms, subsyndromic depression due to circadian rhythm disturbance and fatigue, and higher intrinsic melatonin secretion than normal people. Musical stimulation can activate or increase specific pathways in several brain regions related to emotional behaviour, such as the insular and cingulate cortex^49,50^, hypothalamus, hippocampus, amygdala and prefrontal cortex^51^. Thus, some biochemical mediators, such as increased endorphins^52,53^, endocannabinoids^54^ and dopamine^55–57^ and decreased nitric oxide^58^ regulate positive emotional states^58^. In this study, it was found that, in terms of music selection, the effect of music suggested by experts is better. It may be that experts choose according to the current physiological and psychological state of each patient and the different personality of the patient so as to solve the emotional problems of the patient in a targeted way^59,60^. In terms of intervention cycle, 2–4 weeks is the best effect size for the music intervention cycle^61^, The effect size is moderate when the intervention cycle is less than or equal to 2 weeks, and the effect size is second when the intervention cycle is more than 4 weeks. Qishou Tang also said in the study^62^ that the effect of short-term and medium-term interventions is higher than that of long-term interventions^20,24,30^. However, the reason remains uncleraly, and more high quality RCT to further complement and prove this. The intervention duration ≤ 30 min has a better effect on alleviating depression in breast cancer patients, because with the extension of listening time, the response to two types of auditory stimuli in the human body is different^63^, and the effect of music intervention may be weakened by the timeliness of behavioural habits or distraction of human attention after the intervention duration is higher than 30 min.Intervention duration of 30 min and frequency of 1 time per day had a better effect on depression relief in breast cancer patients^60,61^, which may be related to the timeliness of behavioural habits. It may also be that the frequency of intervention once a day can regulate the stress response of the hypothalamic–pituitary–adrenal axis^64^, which has the effect of regulating the spirit, pleasing the heart, and relieving depression in patients. Similarly, we have observed through research that the course of the disease, family economic conditions and educational background of breast cancer patients may affect their depression, and the type, tone and rhythm of music may also have different effects on the improvement of depression in breast cancer patients. This will inspire some researchers today who can further explore the effects of demographic characteristics and musical elements on depression in breast cancer patients, and look forward to more high-quality RCT articles to further complement and prove.

In summary, based on the literature of high quality controlled trials, each intervention element has different effects on anxiety and depression in breast cancer patients, providing clinical practice and researchers with more precise music intervention programmes. Meanwhile an increasing number of healthcare organisations and government departments are incorporating music therapy into guidelines and policies for breast cancer treatment, and music therapy professionals are being trained and accredited to provide appropriate music interventions for patients. These policy measures help to increase the recognition and acceptance of music therapy interventions by patients and promote the psychological recovery of breast cancer patients. As scientific research continues, we can further understand the specific mechanisms of action and effects of music therapy on patients, and how to better apply music therapy for personalised interventions.

This research has been registered on the INPLASY platform (https://inplasy.com/contact/) under the number INPLASY2023100057.

## Data Availability

The datasets used and/or analyzed during the current study are available from the corresponding author on reasonable request.
